# CD11c-Specific Deletion Reveals CREB as a Critical Regulator of DC Function during the Germinal Center Response

**DOI:** 10.1155/2018/8947230

**Published:** 2018-05-07

**Authors:** Kim Ohl, Anastasia Schippers, Klaus Tenbrock

**Affiliations:** Department of Pediatrics, Medical Faculty, RWTH Aachen, Aachen, Germany

## Abstract

Dendritic cells (DCs) are crucial for the balance between immune response and tolerance, but the molecular mechanism regulating development, differentiation, and homeostasis are poorly understood. The transcriptional activator CREB is involved in regulating different cells of the innate and adaptive immune system and is a transcriptional regulator of development, survival, activation, or proliferation in macrophages, dendritic cells, B cells, and T cells. To directly examine the role of CREB in the regulation of DCs, the *CREB* gene was targeted for deletion with a *CD11c-cre* transgene. The deletion of CREB in CD11c^+^ cells did not involve any developmental or systemic defects within DC populations. However, CREB deficiency in CD11c^+^ cells reduced germinal center (GC) B cells in steady state, and immunization with NP-CGG resulted in a reduced formation of GCs, paralleled by the reduced production of IgGs in sera of immunized mice. In conclusion, we demonstrate that CREB expression in CD11c^+^ cells enhances germinal center responses, most likely by altering DC function, which might have implications for autoimmune diseases that are associated with dysregulated GC responses.

## 1. Introduction

Dendritic cells are professional antigen-presenting cells, which link innate and adaptive immunities by their ability to recognize pathogens and to activate B and T cells. Through their ability to distinguish between self-antigens (Ag) and microbial-associated Ag, DCs play a fundamental role not only in the initiation of immune responses but also in the maintenance of immune tolerance [1]. The activation of DCs involves innate pattern recognition receptors, such as TLRs and NOD-like receptors that recognize microbial associated Ag. Once activated DCs not only present antigens and modulate cell surface costimulatory molecules but also produce cytokines and chemokines, which can be pro- or anti-inflammatory [2]. Immature DCs constantly sample and process Ag. Microbial stimulation and inflammatory conditions induce DC maturation and migration to draining lymphoid organs, where they interact with Ag-specific T cells [3]. The activation of DCs is associated with higher expression levels of costimulatory molecules (CD80 and CD86) and MHC class II (MHC-II) molecules. Classical dendritic cells (cDCs) are found throughout the organism and are equipped with an increased ability to sense tissue injuries, to take up and phagocytose Ag and to subsequently process and present Ag to T cells. Steady-state plasmacytoid dendritic cells (pDCs) show a lower expression of MHC-II, CD11c and costimulatory molecules but produce large amounts of type I IFN upon activation [4]. It is generally assumed that DC maturation status more critically regulates the functionality of DCs than DC lineage alone, but how DCs are regulated to become immunogenic or tolerogenic has not been fully characterized [1, 5].

The transcriptional activator CREB belongs to the superfamily of bZip proteins. Members of this family of proteins contain a basic domain/leucine zipper domain, which binds to an 8-base pair palindromic DNA sequence, called CRE site [6–8]. CREB is activated by several protein kinases such as PKA, CAMKII, CAMKIV, MAPK, MSK1/2, and PKC, while Ser/Thr phosphatases, protein phosphatase 1 (PP1) and PP2A negatively regulate CREB [9]. The activation of CREB is induced by phosphorylation within the CREB kinase inducible domain (KID) and recruitment of CBP (CREB binding protein)/p300. With regard to the immune system, CREB is a transcriptional regulator of development, survival, activation, or proliferation in macrophages; dendritic cells; B and T cells; and controls cytokine production from both innate and adaptive immune cells, including IL-10, IL-23, TNF-*α*, IFN-*γ*, and IL-2 [9–13].

CREB is directly involved in signaling pathways that regulate DCs. The activation of MAPK is crucially involved in the regulation of DC maturation, survival, and cytokine secretion [14, 15]. Importantly, the p38-MAPK pathway critically regulates DC costimulatory receptor expression, T-cell proliferative capabilities, and cytokine production as well as multiple genes involved in DC maturation [16]. Interestingly, major transcription factors that lie downstream of the p38-MAPK pathway are CREB and activating transcription factor 1 (ATF1) [17, 18]. Recent studies describe a potential role for p38-MAPK-CREB signaling in DCs [13, 19, 20]. The p38-MAPK-CREB axis is associated with the upregulation of costimulatory molecules [19, 20] and the secretion of IL-10 [13]. Thereby, CREB signaling in DCs might engage distinct T-cell programs. Armbruster et al. could show that the p38–CREB–IL-10 axis is one molecular mechanism for DC tolerogenicity and regulatory T cell (T_reg_) differentiation, which is induced by PSM*α* peptides in vitro [13]. On the other hand, Li et al. recently showed that an MST1-p38MAPK-MK2/MSK1-CREB pathway is a critical signaling pathway in DCs, which controls IL-6 production and IL-6R-STAT3 expression in CD4^+^ T cells and thereby directs T_h17_ cell differentiation [21].

Although several studies suggest a key role of CREB in DC maturation and function, a detailed lack of function study in vivo is still missing. We therefore deleted CREB in CD11c cells and analyzed DC populations and functions in these mice.

## 2. Methods

### 2.1. Mice

Experiments were performed with age-matched C57BL/b WT (CREB-flox) and CKO (CD11c-CRE × CREB-flox) and OT-II mice. CKO mice were generated by crossing CREB-flox mice (kindly provided by G. Schütz [22]) with CD11c-CRE mice [23], and CREB-flox mice were used as WT controls. All mice were bred in our animal facility and kept under standardized conditions. The study was approved by the regional government authorities, and animal procedures were performed according to German legislation for animal protection. The permission for the projects has been granted by the Regierungspräsident/LANUV Nordrhein-Westfalen.

### 2.2. Cell Isolation

Mouse BM cells were flushed from femurs and tibias with Dulbecco medium. Erythrocytes were lysed with lysis buffer (eBioscience) for 3 min at room temperature, and the remaining cells were washed once with PBS. Single-cell suspensions were isolated from spleens, pLNs, and thymi, and erythrocytes were lysed with lysis buffer and washed once with PBS. CD4^+^ cells were isolated by magnetic cell separation using the CD4^+^ T cell isolation kit (Miltenyi).

### 2.3. In Vitro DC Generation

DCs were generated by culturing BM cells in RPMI medium supplemented with 1% penicillin/streptomycin, and 10% inactivated fetal calf serum (Gibco Life Technologies, Germany) in the presence of GM-CSF (50 ng/ml) and IL-4 (40 ng/ml) for 6 days.

### 2.4. T Cell Coculture Assays

DCs were generated by culturing BM cells in the presence of GM-CSF (50 ng/ml) and IL-4 (40 ng/ml) for 6 days. Cells were fed with ovalbumin (OVA) peptide (1 *μ*M) for 2 hours and extensively washed with PBS. CD4^+^ OT-II cells were isolated by magnetic cell separation and labeled with cell proliferation dye (5 *μ*M) (eBioscience) according to manufacturer's instructions. DCs and CD4^+^ T cells were cocultured in 1 : 10 ratio in U-bottom 96-well plates.

### 2.5. Flow Cytometry

For surface staining, single-cell suspensions were stained with anti-PDCA1, anti-CXCR5 (BD Biosciences), anti-CD11c, anti-CD86, anti-CD80, anti-MHC-II, anti-CD4, anti-CD3, anti-CD8, anti-CD25, anti-CD19, anti-B220, anti-GL-7, and anti-ICOS antibodies (all from eBioscience, Germany). To analyze Foxp3, cells were fixed and permeabilized with FOXP3 staining buffer set (eBioscience, Germany) following the manufacturer's instructions and stained with anti-Foxp3 antibodies (eBioscience, Germany). Flow cytometry was carried out using FACSCanto II device (BD Biosciences, Germany). Data analysis was performed using FCS Express Software.

### 2.6. ELISA

For the detection of NP-specific antibodies in the serum of immunized mice, 96-well plates were coated overnight with NIP (10 *μ*g/ml) in BSA, blocked for 2 hours, and incubated with sera (serial dilutions starting at 1 : 50). After washing, the serum antibodies adherent to plate-bound NIP were detected by HRP labeled anti-mouse IgG antibodies (Jackson ImmonoResearch, USA) and o-phenylenediamine. The reaction was stopped by using 1 N HCL. Plates were read at a wavelength of 492 nm. For the detection of total IgG levels, murine IgG ELISA Kit (Mabtech) was used according to the manufacturer's instructions.

### 2.7. Statistical Analysis

All data are presented as mean ± standard error (SEM). Differences between the two groups were evaluated using two-tailed unpaired or paired (if indicated) Student's *t*-test if data were normally distributed. Otherwise, a non-parametric Mann–Whitney test was performed. All statistical analysis and subsequent graphics generation were performed using GraphPad Prism version 7.0 (GraphPad Software, USA). A *p* value < 0.05 was considered to be statistically significant.

### 2.8. Immunization

For T-cell-dependent immunization, groups of age-matched mice were immunized i.p. with 100 *μ*g 4-hydroxy-3-nitrophenylacetyl chicken gamma globulin (NP-CGG) (Biosearch Technologies, USA) in Imject Alum (Thermoscientific, USA). Spleens were harvested 10 d later.

### 2.9. Immunohistochemistry

For immune fluorescence, spleens were harvested and immediately frozen in embedding (tissue-tek; Sakura, NL) media. Sections were cut in 7 *μ*m of thickness. CD3 was detected using CD3e APC antibody (eBioscience, USA). To detect GCs, sections were stained with biotin conjugated PNA (Vector Laboratories, USA) followed by DyLight488 conjugated streptavidin (Thermo Scientific, USA), and sections were mounted with Fluoromount-G (Southern Biotech, USA). Images were captured using a Cool SNAP HQ2 camera and an Axioplan 2 Imaging microscope (Zeiss, Germany) with VisiView software.ays later.

## 3. Results

### 3.1. DC Homeostasis Is Not Affected by CREB Deficiency in Steady State

**Figure 1:**
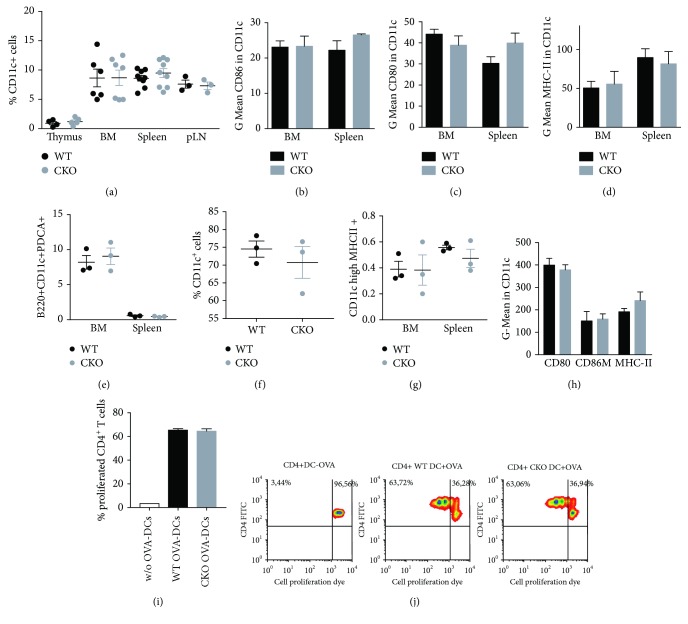
Steady-state DC homeostasis is not affected by CREB deficiency: (a) percentages of CD11c^+^ cells in different lymphoid organs of indicated mice determined by flow cytometry; (b–d) activation marker expression in CD11c^+^ cells from WT and CKO mice (*N* = 3 each; mean fluorescence intensity was calculated with error bar representing SEM); (e) percentages of cDCs of indicated mice determined by flow cytometry; (f) percentages of pDCs of indicated mice determined by flow cytometry; (g) percentages of CD11c-expressing cells after the in vitro differentiation of BMDCs with GM-CSF and IL-4 for 7 days; (h) activation markers in CD11c-expressing cells after the in vitro differentiation of BMDCs with GM-CSF and IL-4 for 7 days (mean fluorescence intensity was calculated with error bars representing SEM). (i) Differentiated BMDCs of WT or CKO animals were either left untreated or were pulsed with OVA peptide (1 *μ*M) for 2 h and extensively washed with PBS. BMDCs were cocultured with cell proliferation dye labeled T Cells of OVA-transgenic OT-II mice. FACS analysis of T cells was performed after 3 days. The number represents percentage of proliferated cells in each quadrant (*N* = 3). (j) Representative density plot of (i). For (a, e, f, and g), each dot represents an individual mouse, and horizontal bars indicate SEM.

To analyze the effect of CREB on DC homeostasis, we first examined percentages of DCs and DC subsets within bone marrow (BM) and peripheral lymphoid organs. CREB deletion in CD11c^+^ cells did not alter the percentages of CD11c^+^ cells within thymus, BM, spleen, or pLNs (Figure 1(a)). Furthermore, the expression of activation markers such as CD86, CD80, and MHC-II was comparable to WT CD11c^+^ cells (Figures 1(b)–1(d)). Moreover cDC and pDC populations were not affected by CD11c-specific CREB deletion (Figures 1(e) and 1(f)). To analyze whether the absence of CREB affects the DC development in vitro, we cultured WT and CKO BM cells in vitro with GM-CSF and IL-4 for 6 days. No differences were observed within the ratio of in vitro differentiated DCs (Figure 1(g)), and again, the expression of activation markers was not altered (Figure 1(h)). However, one has to keep in mind that during in vitro DC development, a relatively late onset of *Cre recombinase* occurs, so that the role of CREB in DC differentiation cannot be excluded. To analyze the capacity of in vitro-generated DCs to activate T cells, we performed antigen-specific T-cell proliferation assays. OVA-fed DCs from WT and CKO mice showed a similar ability to the enhance proliferation of OT-II CD4^+^ T cells in vitro (Figures 1(i) and 1(j)). In summary, we found a normal DC compartment in CKO mice, with no developmental or systemic defects.

### 3.2. CREB Deletion in DCs Reduces Spontaneous B Cell Activation

**Figure 2:**
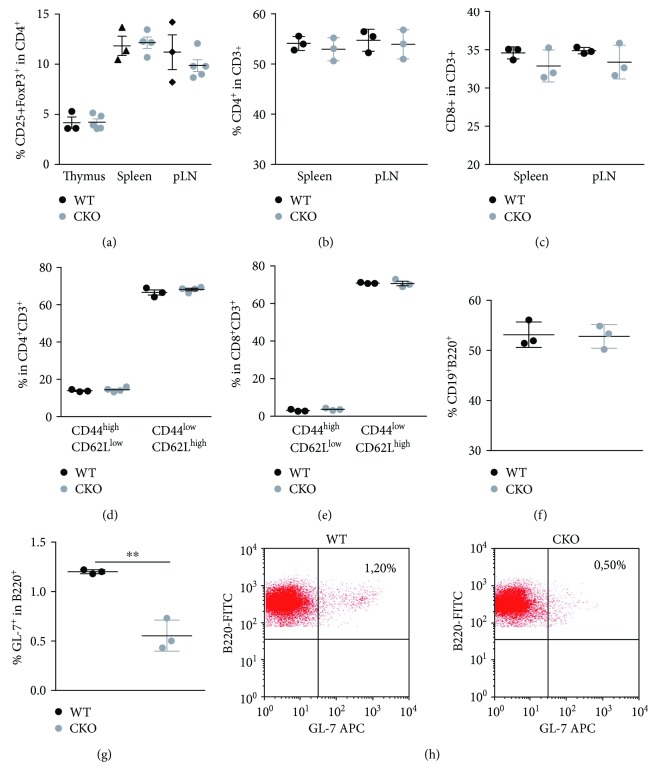
CREB expression in DCs is not necessary to preserve T-cell homeostasis but alters B cell activation: (a) percentages of CD25^+^Foxp3^+^ cells within CD4^+^ T cells in different lymphoid organs of indicated mice determined by flow cytometry; (b) percentages of CD4^+^ within CD3^+^ T cells in spleens and pLNs of indicated mice determined by flow cytometry; (c) percentages of CD8^+^ within CD3^+^ T cells in spleens and pLNs of indicated mice determined by flow cytometry; (d) percentages of naive (CD62L^high^, CD44^low^) and memory (CD44^high^, CD62L^low^) cells within CD4^+^ T cells; (e) percentages of naive (CD62L^high^, CD44^low^) and memory (CD4^high^, CD62L^low^) cells within CD8^+^ T cells; (f) percentages of CD19^+^B220^+^ cells in spleen of indicated mice; (g) percentages of GL-7^+^ cells within CD19^+^ cells, unpaired, two-tailed Student's *t*-test, *p* = 0.0021; (h) representative dot plots showing GL-7^+^ cells within B220^+^ population. For (a–g), each dot represents an individual mouse, and horizontal bars indicate SEM. ^∗∗^*p* < 0.01.

DCs are important for maintaining peripheral T-cell homeostasis and preventing inappropriate T-cell activation; furthermore, they are also involved in thymic T-cell development. The self-antigen presentation by thymic DCs can result in the negative selection or lead to the generation of thymus-derived T_regs_ [20]. Nevertheless, percentages of T_reg_, CD4, and CD8 cells and effector and memory ratios within CD4 and CD8 population were not different in lymphoid organs in steady state (Figures 2(a)–2(e)). However, we found striking differences within the B-cell population. The percentages of overall B cells were not altered (Figure 2(f)), but percentages of spontaneous germinal center (GC) B cells were reduced (Figures 2(g) and 2(h)). This prompted us to analyze if the deletion of CREB in CD11c^+^ cells influences GC formation after immunization with NP-CGG.

### 3.3. CREB Expression in DCs Contributes to Efficient GC Formation

**Figure 3:**
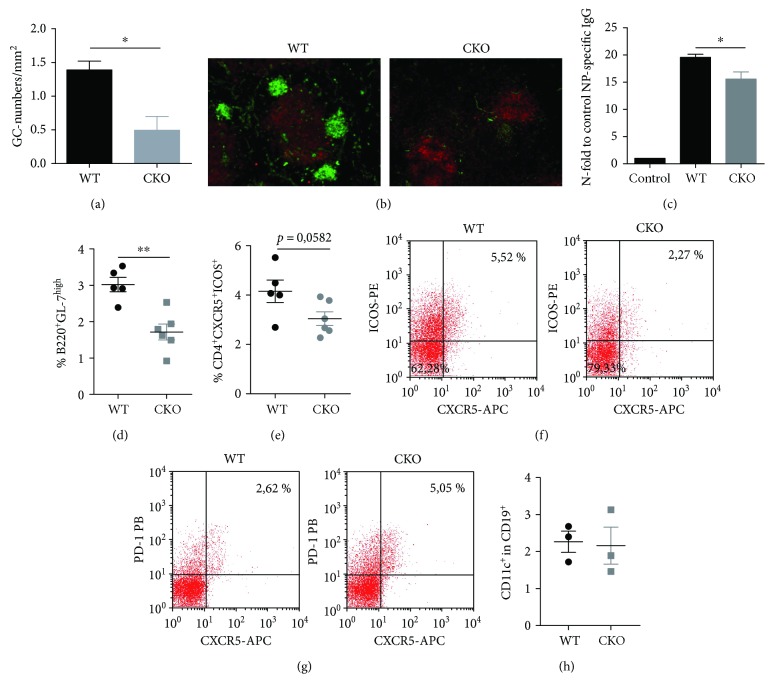
Enhanced GC responses in CKO mice: (a) statistical analysis of frozen spleen sections stained with anti-CD3-APC antibody and biotinylated PNA/streptavidin DyLight-488. The number of GCs per mm^2^ spleen was counted in WT and CKO mice after immunization with NP-CGG and Alum (*N* = 3, unpaired, two-tailed *t*-test, *p* = 0.0212). (b) Representative frozen spleen sections from immunized mice stained with anti-CD3-APC antibody (red) for T cells and biotinylated PNA/streptavidin DyLight-488 (green) for GC cells. The slides were imaged with a 10x objective using a fluorescence microscope. (c) ELISA, using plates coated with NIP-BSA for analysis of anti-NP-IgGs. Anti-NP antibodies were detected with anti-mouse IgG. Data indicate the *n*-fold upregulation of OD values compared to OD-values of unimmunized control mice (mean percentage ± SEM of 5 animals, two-tailed, unpaired *t*-test, *p* = 0.0241). (d) Percentages of GL-7^+^ cells within B220^+^ cells in spleens of immunized mice determined by flow cytometry (two-tailed, unpaired *t*-test, *p* = 0.0018); (e) percentages of CXCR5^+^ICOS^+^ cells within CD4^+^ T cells in spleens of immunized mice determined by flow cytometry; (f) representative dot plot showing T_fh_ cells (as ICOS and CXCR5-expressing cells within CD4^+^) in WT and CKO mice after immunization; (g) representative dot plot showing T_fh_ cells (as PD-1 and CXCR5-expressing cells within CD4^+^) in WT and CKO mice after immunization; (h) percentages of CD11c^+^ cells within CD19^+^ cells in spleens of immunized mice determined by flow cytometry. For (d, e, and h),each dot represents an individual mouse, and horizontal bars indicate SEM. ^∗^*p* < 0.05 and ^∗∗^*p* < 0.01.

To further determine whether CREB affects the innate DC function and thereby regulates adaptive immune responses, we made use of a T-cell-dependent immunization model. GCs were evaluated 10 days postpriming with NP-CGG and alum. The average number of GCs in spleens of CKO mice was about 2-fold lower than that in WT spleens (Figures 3(a) and 3(b)). Consistent with this, NP-specific IgGs in sera of mice were reduced in CKOs as well (Figure 3(c)), and frequencies of GC B cells were clearly reduced in the absence of CREB in CD11c^+^ cells (Figure 3(d)). Follicular T helper cells (T_fh_) in GC provide direct help to antigen-specific B cells [24]. The percentages of T_fh_ cells were tendencially decreased in CKO mice as well (Figures 3(e)–3(g)). For the CD11c-CRE mice, *Cre* expression has been shown in subsets of T, B, and NK cells, and recent studies show a central role for CD11c^+^ B cells that arise in autoimmune-prone conditions and drive the development of autoimmunity [25, 26]. We therefore also analyzed numbers of CD19^+^CD11c^+^ cells in WT and CKO mice after immunization, however we did not detect any differences between both groups (Figure 3(h)).

We concluded from this that the CREB expression in CD11c^+^ cells is important for antigen-specific B-cell responses within the GC reaction, probably by regulating DC function.

## 4. Discussion

Despite the critical role of DCs in the immune response, molecular mechanisms directing their development, subset specification, and homeostasis are only partially understood. DC development is known to be controlled by several important transcriptional regulators, among others, NF-*κ*B subunits, Ikaros, PU.1, Id2, interferon regulatory factor (IRF) 8, RBP-J, and STAT3 [23, 27, 28].

A crucial role for CREB in dendritic cells has been supposed since long ago. Especially, the p38 MAPK-CREB/ATF1 is known as an important pathway for DC activation and DC maturation [13, 20]. Furthermore, studies indicate a critical role for CREB as the inducer of IL-10 production in dendritic cells in response to saccharomyces-derived zymosane or *Staphylococcus aureus*-derived PSM peptide [12, 13]. Nevertheless, until now, *in vivo* studies with a CD11c-specific deletion of CREB were missing.

Within this study, we show that CREB is generally dispensable for DC lineage commitment and does not alter DC homeostasis in steady state. As the here used *CD11cCre* is mainly expressed in mature and terminally differentiated DCs, we cannot fully exclude that CREB is involved in former DC differentiation processes. However, the CREB expression in CD11c^+^ cells emerged as an important contributing factor for a regular GC response, most likely in a DC-dependent manner. However, one limitation of our study is that the CD11c-specific deletion of CREB might also target CREB in other immune cells. For CD11c-CRE mice, *Cre* expression has been shown in the subsets of T, B, and NK cells, as well as in some monocyte and macrophage populations [23, 29, 30]. Until now there are very few lines for DC targeting available, and to our knowledge, no line that specifically deletes genes in all DCs exists. Experiments with CD11c–CRE × ROSA-EYFP mice show a very efficient deletion in cDCs and pDCs, which makes CD11c-CRE mice a suitable tool to study CREB deletion in both DC subtypes; however, we have to consider that aside from DCs, other cell populations are targeted as well. Especially, CREB deletion in T and rather B-cell subsets might contribute to our observations, as B cells show a spontaneous activation in CKO mice. In this context, it is interesting that CD11c-expressing B cells located at the T cell/B cell border in the spleen are potent APCs and promote the development of autoimmunity [25, 31]. We did not find altered numbers of these cells in our CKO mice but cannot exclude functional alterations. However, due to the central role of DCs within the GC response and the low numbers of CD19^+^CD11c^+^ cells in our mice, we would speculate that our observations result primarily from alterations within DCs, but more specific knock-out models are required to strengthen this hypothesis.

GCs not only form in the B cell follicles of secondary lymphoid tissues during infection and immunization with foreign antigen [32] but are also common in murine models of lupus [33–36] and occur frequently in patients with autoimmune diseases such as arthritis and SLE, where their presence correlates with the production of pathogenic autoantibodies [37, 38]. Therefore, modulating humoral immune responses—T_fh_ dependent or independent—is a key step to regulate defects in humoral immunity. CKO mice revealed reduced percentages of T_fh_ cells as well, which suggests that CREB deficient DCs are less capable of antigen uptake and presentation or effective stimulation of T_fh_ cells within the germinal centers. This could be explained by a maturation defect of CREB-deficient DCs or by a reduced production of cytokines. Within GCs, follicular dendritic cells (FDCs) are essential for the maintenance of GCs. They not only retain antigen for extended periods but furthermore secrete cytokines, such as IL-6 and B-cell activating factor (BAFF), and thereby promote B-cell differentiation and survival [39]. Most interestingly, a quite recent publication identified a new role for FDCs. By sensing self-antigen DAMPs with subsequent secretion of IFN-*α*, they enhance autoreactive GCs and autoantibody titers [40]. Therefore, FDCs and their regulation are an exciting area of further studies and are promising targets in autoimmune diseases. If and how CREB is involved in regulation of FDCs will be a matter of further studies.

## 5. Conclusions

Our study suggests that the CREB expression in CD11c^+^ cells is involved in T_fh_ and GC B-cell differentiation and that this most likely results from an altered DC function in the absence of CREB. As enhanced GC responses are associated with the development of autoreactivity in vivo, the further analysis of the CREB-mediated mechanism in CD11c^+^ cells during GC reactions might uncover new therapeutic options.

## Abbreviations

Ag: Antigen
CKO: Conditional knock-out
DC: Dendritic cell
GC: Germinal center
NP-CGG: 4-Hydroxy-3-nitrophenylacetyl chicken gamma globulin
OVA: Ovalbumin
Tfh: Follicular T helper cell
Treg: Regulatory T cell
WT: Wildtype (CREB-flox).
